# Use of the sun as a heading indicator when caching and recovering in a wild rodent

**DOI:** 10.1038/srep32570

**Published:** 2016-09-01

**Authors:** Jamie Samson, Marta B. Manser

**Affiliations:** 1 Department of Evolutionary Biology and Environmental Studies, University of Zurich, Switzerland

## Abstract

A number of diurnal species have been shown to use directional information from the sun to orientate. The use of the sun in this way has been suggested to occur in either a time-dependent (relying on specific positional information) or a time-compensated manner (a compass that adjusts itself over time with the shifts in the sun’s position). However, some interplay may occur between the two where a species could also use the sun in a time-limited way, whereby animals acquire certain information about the change of position, but do not show full compensational abilities. We tested whether Cape ground squirrels (*Xerus inauris*) use the sun as an orientation marker to provide information for caching and recovery. This species is a social sciurid that inhabits arid, sparsely vegetated habitats in Southern Africa, where the sun is nearly always visible during the diurnal period. Due to the lack of obvious landmarks, we predicted that they might use positional cues from the sun in the sky as a reference point when caching and recovering food items. We provide evidence that Cape ground squirrels use information from the sun’s position while caching and reuse this information in a time-limited way when recovering these caches.

Many animals use cues to orientate, whether it is following simple concentration gradients^1^ or using celestial cues such as stars^2^. With an elliptic daily movement across the sky, the sun is suggested to be one of the most dominant cues used by diurnal species, providing a frame of reference throughout the day^3^. Animals may use the sun as an orientation cue when navigating during both local^4^ and/or more widespread movements^5^. One difficulty with using such a celestial body is that animals need to be able to demonstrate time compensation, allowing them to account for the continued shift of the sun in the sky as the earth rotates^3^,^4^. For example, homing pigeons (*Columbia liva*) have a highly developed sun compass and can account for these shifts, possibly through the perception of the arc of the sun’s movement^6^,^7^. Where the shifting position of the sun is not controlled for, some species may use the sun in a time-dependent manner, using fixed positional information from the sun^7^. However, there appears to be some interplay between both mechanisms, with some species using these cues in a time-limited manner^3^. This mechanism makes use of specific information that is then reused in a partially flexible way, but full time compensational abilities that account for the sun’s shifting position are not present^3^.

For centuries, human navigators have used the sun as a compass relying on its azimuth angle, which is the angle of the sun in relation to a fixed reference, such as true north. The sun’s azimuth can be used in two distinct ways, either as a compass^8^, where it indicates the absolute direction with respect to the earth’s surface or as a heading indicator, giving an animal a general bearing to set its movement by^9^. Within familiar areas, individuals may be expected to use the sun as a heading indicator instead of a compass, as solar information is often used in conjunction with habitat features^10^, where the absolute direction with respect to a global position is not required. In these species, the sun may be used even with the availability of additional landmark information^9^. Cue use is suggested to show a heterarchy^11^,^12^, where one cue is predominantly favoured over others during different types of orientation and navigation movements. Rather than making all other cues redundant, animals may collate information, utilising a number of cues which is known as the “multiple bearings hypothesis”^13^. This hypothesis postulates that animals can increase the accuracy of their orientation by combining directional information from a number of sources. In Clark’s nutcrackers (*Nucifraga columbiana*), for example, it has been suggested that through use of bearings from multiple landmarks they can reduce any error they may acquire from directional information estimated solely from the sun^13^. Rather than making cues from the sun redundant and favouring landmarks cues, diurnal species may still use celestial information due to its saliency^4^.

As the sun can provide directional information, this cue can be useful for caching animals when depositing and relocating food items. There have been a number of studies to suggest that some bird species^14^,^15^ may use cues from the sun during caching events, but there are no examples of mammals explicitly using the sun in this context. Most of the caching studies have concentrated on determining whether animals use the sun through clock-shift experiments^14^,^15^, but how animals use the sun is under debate. Although it is generally accepted that the sun compass can be used in a flexible way, i.e. full time compensation^3^,^4^,^6^,^16^, less is known about the degree of flexibility when the sun is used as a heading indicator. For example, individuals can use a heading indicator to orientate to a location as well as accounting for shifts in this indicator to reorientate back to the same location. Clark’s nutcrackers^17^ and rats^18^ (*Rattus norvegicus*) show some flexibility in finding a reward when landmark arrays are rotated whilst keeping the configuration constant. This suggests that animals may be treating landmark arrays as units of information, identifying the position of a reward within the array. Although only landmark arrays were used in these examples, the sun can potentially act as a point within a unit of an array, or it can act as a heading indicator in which to calibrate other information against^9^. Furthermore, animals may show some flexibility in the use of this information if they are able to account for the movement of the sun in relation to landmark features. This can arise through simple associative learning processes, whereby individuals learn the relationship of the sun and the “visual scene”, within familiar areas^3^.

In the Kalahari Desert, Southern Africa, where cloud cover is sporadic, the sun is a prominent feature and is continually visible throughout the diurnal period (~3730 hours of sunshine a year, global average 2334 hours, source WDC for Meteorology). In this study, we predicted that Cape ground squirrels (*Xerus inauris*), a social rodent species, use the sun as a frame of reference when navigating in the context of caching and recovering food items. We provisioned subjects with food at various points within their home range, at four time intervals throughout the day, to determine directional patterns in caching placement behaviour. When subjects moved from the provisioning point to the cache point, we took GPS points of the track they made. From these data we were able to calculate various track properties, such as its angle in relation to the sun and the straightness. In addition, we examined recovery behaviours to determine the role the celestial system might play when subjects re-orientated back to food they had previously cached.

## Methods

### Study site and species

The study was undertaken at the Kuruman River Reserve in South Africa (26°58′ S, 21°49′ E), at the site of the Kalahari Meerkat Project. The reserve is located in the green Kalahari, a semi-arid region of sand dunes and flat terraces, with sparse vegetation^19^. Cape ground squirrels inhabited the site^20^ and formed colonies that were dispersed along the dry Kuruman riverbed. This species is a social central place foraging rodent that lived in groups of up to 36 individuals (the largest recorded at the reserve since the study started in 2011). These groups comprised adult males and females (with a female bias) and a number of sub-adults, juveniles and pups of both sexes^20^. Most individuals were habituated to close human observation, allowing us to follow individuals within less than 1 m whilst they foraged. Additionally, all individuals were uniquely marked using black hair dye (L’Oreal, Garnier) so that they could be individually recognised. The procedures performed in this study were approved by the University of Pretoria Animal Ethics Committee (permit number: S4532-13). All methods were carried out in accordance with the approved guidelines in this permit. In addition, Northern Cape Nature Conservation granted research permits for the study (number: ODB #1486/2013).

### Data collection

Data was collected between April and July 2014 on 9 adult Cape ground squirrels across 5 social groups. Before each observation was initiated, the observer visited a social group to locate the study subject. The subject was then provisioned with a single peanut in the shell and a GPS point was taken (Garmin GPS MAP62 handheld GPS device, Garmin LTD, 1996–2013, radial standard accuracy of ±1.5 m). This provisioning point was defined as the start point of the subject’s track (hereafter, start point). The provisioned food items were standardized (2.5 cm long peanuts, in the shell) to reduce variation in behaviour caused by food of varying quality. When the subject moved to the cache site, GPS points were taken every time the subject showed any deviation from a straight path and when the subject cached, a final GPS point was taken (the caching location was defined as the end point of the tracks, hereafter, stop point). For each observation period, subjects were provisioned with 5 nuts (in successive presentation trials after the previous nut had been cached, typically within 5 min) following the same protocol as described above. Subjects were observed at 4 different time periods per observation day, so that any effect of time of day was controlled for. These observation periods were determined by dividing the time between sunrise and sunset by 5, giving 4 time points with start times of (mean ± sd): 8:46:46 (h:m:s) ±5.52 minutes, 10:59:13 ± 1.46 minutes, 13:11:40 ± 4.06 minutes, and 15:24:07 ± 8.41 minutes. As the sunrise and sunset times constantly change, these periods were calculated for each day, enabling us to conduct observations at the same relative time each day. As the ground squirrels got up after sunrise and went below for the evening before sunset, these solar periods could not be examined. For each of the 9 study subjects, 3 repeats were attempted for each time period. For some subjects we missed observations due to them not being present during the period, either because the subject was below ground (for the 1^st^ observation period) or it could not be found.

### Calculation of track angles

**Figure 1:**
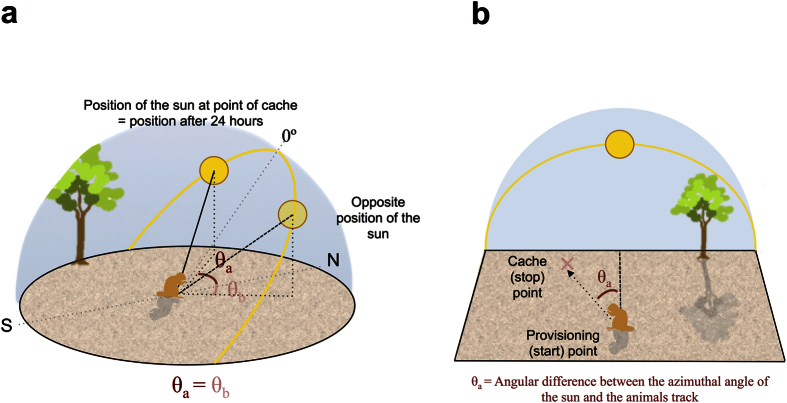
A diagrammatic representation of how the track angles were calculated (see also Supplementary Fig. S2). (**a**) An illustration of the path of the sun through the sky and how the azimuthal (and elevation) angles are the same when you consider opposite positions of the sun on its arc. (**b**) Any caches recovered close to the 24-hour point (θ_a_) were defined as being recovered within the A24 hr category. For caches that were recovered closer to this opposite azimuthal angle of the sun (θ_b_), they were defined as being recovered within the PRE24 hr category.

Initially for each of the 5 groups, a hypothetical caching area boundary was determined using the distance between the centre of the home burrow and the cache point furthest away from it as the radius length for the circle that defined the caching area. The angle of the sun (hereafter, the azimuthal angle) in relation to the centre of the burrow was extrapolated to this boundary line and the coordinates calculated. The azimuthal angle was calculated using data obtained from SunEarthTools (www.sunearthtools.com, see Supplementary Fig. S1 for an example). The position of the sun was calculated for the exact date and time of the tracks when caching, using coordinates of the centre of the home burrow associated with each study subject to obtain these measurements. The track angles were then calculated by extracting the angular difference (in radians) between the cache track and the azimuthal angle (Fig. 1a and see Supplementary Fig. S2).

### Statistical analysis

All statistical analyses were performed in R; release GUI 2.1^21^. Linear mixed effects models (hereafter, LMM^22^) were performed using the “lme4” package in R for all models as this allowed us to include random effects (see Supplementary Table S1 for model structures and summaries). To control for variation in these parameters, we included subjects nested within group as random factors. To determine the significance of fixed effects, we used likelihood ratio tests (hereafter, LRT^23^) to compare LMMs with and without these effects. For comparisons of distributions, we used Kolmogorov-Smirnov tests (hereafter, KS-test).

#### Distribution of track angles in relation to the sun

The raw data for angular differences between the tracks and the sun were transformed, so that all values were in relation to 0 radians (analogous to the azimuthal angle of the sun when the track was recorded). This transformation was achieved by taking the azimuthal angle between the track and the sun and scaling these angles in relation to zero, so that tracks to the right of the sun (0 radians) were given positive radian values and to the left, negative values. As individuals also moved away from the sun during caching events, we transformed the angles of these tracks so that values were also relative to 0 radians, by reflecting these angles about the line connecting −1/2π radians and 1/2π radians. In doing so, the data were bounded between −1/2π radians and 1/2π radians. By analysing the data in this way, circular statistics^24^ were avoided, as we did not need to control for issues arising from data being on a true angular scale. In addition, as we recorded more than one track per subject, post-hoc mixed modelling allowed us to control for repeated measures, deal with the unbalanced design and control for subject and group variation. A dip test (“diptest” package^25^) was performed on the distribution of raw data to determine the number of modes, which suggested the distribution of angles showed non-unimodality. We then ran a finite mixture model using an expected maximisation (EM) algorithm to determine parameter estimates for the components of the multimodal distribution observed (“mixtools” package^26^). To extract the standard error (SE) for each parameter estimate, we bootstrapped (B = 2000) the mixture model^27^. This provided us with SE estimates for the lambda (proportion of overlap of the two distributions), variance and mean.

The component distributions extracted after running a finite mixture model described above were compared using KS-tests and LMM’s. To compare distributions, we examined the differences by inverting the values of the distribution where tracks were made to the left of the sun (at negative radian angles), which put both on the same scale. We then ran a KS-test on the data to determine if there were overall differences in these two component distributions and then ran an LMM to determine whether there were differences in the size of track angles between the two. Finally, we ran LMMs to identify if the angle values for each distribution were significantly different from zero (i.e. the angle of the sun).

#### Examining across observation periods

To determine whether the track angles changed with the shifting angle of the sun, we ran a LMM, with the absolute angle of the sun as a fixed effect. As a response, we used the track angle in relation to the angle of the sun at a given time point. If subjects were using the sun as a heading indicator similarly across observational time periods, then the azimuthal angle difference between the sun and track should remain constant, as the sun moved along its arc of trajectory.

#### Track straightness

We used the “move” package^28^ in R to determine the track length made by a subject when moving from the start to the stop points during caching. The Euclidean distance between the start and stop points was then calculated, which gave us the shortest distance between the two points. After which, we calculated the ‘straightness index’^24^, which is the Euclidean path length divided by the observed path length, where a value of 1 would suggest a track was straight and 0 where a track was highly tortuous.

### Recovery success and time

In addition to using the sun as a heading indicator when caching, we investigated if caches were recovered using cues from the sun. Due to logistical issues we were unable to record the tracks of the animals during cache recovery. Therefore, we calculated the difference between the azimuthal angles of the sun at caching and recovering as a proxy for the reuse of solar information from cache events. The reason for doing so was that if caches were recovered at similar azimuthal angles to the sun as that at the cache event, the resulting pattern would suggest subjects were reusing solar information rather than alternative cues such as landmarks. We examined the unearthing of caches (n = 47) by setting up remote cameras (5210A series, LTL-Acorn Outdoors) near the cache site for 5 days and recorded 15-second videos whenever the infrared component was triggered. This allowed us to calculate the time lag between caching and unearthing for both stolen caches and those recovered by the cacher. For recovered caches (n = 20), we calculated the azimuthal angles of the sun in the sky when the caches were made and when they were recovered. As the time and location of the recovered cache was known, we were able to calculate the accurate local azimuthal angles of the sun for the recovery event using the method described previously for the azimuthal angles at caching. Due to the fact that this is a social species, there is a high risk of caches being stolen (87.5% of caches that were stolen were done so within 24 hours (n = 24) and 57.4%, of caches were stolen in total (n = 27)) and therefore subjects may recover within 24 hours to reduce the likelihood of cache theft. As a number of caches were recovered within 24 hours (n = 12, 60%, overall caches had a mean recovery time of 16.03 ± 5.84 hours, mean ± sd), we hypothesised that subjects may show some flexibility in their use of the sun, but not show full time compensation.

As the sun’s orbit along the arc of trajectory is symmetrical about the North-South plane (the sun’s zenith), the azimuthal angles of the sun therefore also show symmetry (Fig. 1b). We compared the azimuthal angle of the sun in relation to the centre of the group’s burrow at recovery to both the azimuthal angle at 24 hours after the cache and the opposite azimuthal angle to this 24-hour point (Fig. 1b). Any caches that were recovered closer to this opposing rather than the 24-hour position were categorised as being recovered at the pre-24 hour point, hereafter PRE24 hr. Any caches that were recovered closer to the 24-hour point were categorised as being recovered at the 24-hour point, hereafter A24 hr. Using this data, we were able to examine the relationship between the recovery and cache azimuthal angles of the sun using LMMs, with the recovery angle as the response and cache angle as a fixed effect. The differences between azimuthal angles of the sun at caching and recovering were extracted to determine how accurate subjects were at recovering caches at the PRE24 hr and the A24 hr categories at varying elevation angles of the sun. Furthermore, we examined this difference to determine what the effect of the sun’s elevation at the point of caching had on recovery accuracy.

### Foraging areas

The areas of the start points were calculated for each subject by identifying the minimum convex hull of these points (“alpahull” package^29^). The areas of the hulls were determined by setting the alpha value of the hull at the minimum value where all boundary points were covered. The area was then extracted using an inbuilt package function. A null foraging area was determined by calculating the maximum distance at which start points were recorded from the centre of the burrow for each subject and using this as the radius to calculate the area (mean ± sd, 34.4 ± 29.7 m). If subjects homogenously distributed themselves throughout their home range, the area of the convex hulls should not differ from that of the null area estimate.

## Results

### Track angle patterns

**Figure 2:**
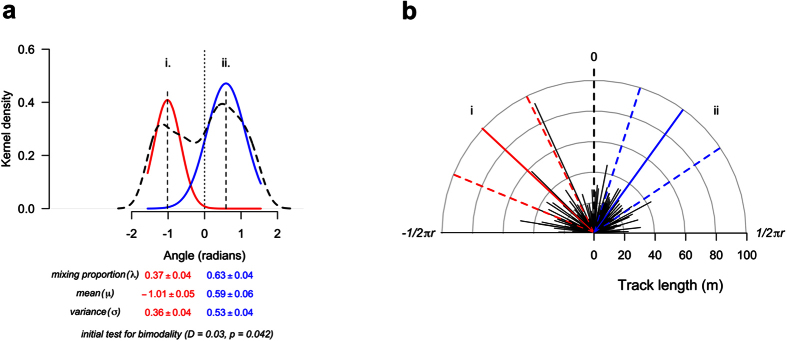
The azimuthal angle between the tracks and the sun for each of the recorded cache track events. Observed bimodal distribution of track angles from the sun (dashed line, N = 265) and two distributions extracted using a finite mixture model (two-component distributions were identified, red (i.) and blue (ii.), (**a**) An angle of 0 radians would mean the subject moved directly towards or away from the sun. The values beneath the plot represent bootstrap estimates of the standard error for each summary statistic. Visualisation of how the track angles (mean ± sd, solid and dashed coloured lines respectively) of the two mixture distributions differed from the angle of the sun (represented as 0, (**b**)). Straightened observed track angles and lengths are shown in black.

The angles of the tracks in relation to the sun were plotted and the distribution of angles showed bimodality (Hartigans’ dip test; D = 0.03, *P* = 0.042, Fig. 2a). As no difference was found in the distribution of track angles depending on whether subjects moved towards or away from the sun (KS test; D = 0.10, *P* = 0.760), we pooled data to model absolute track angles. This lack of difference between the two previously described distributions further justified why these results could be analysed without the need for circular statistics. We found that the component distributions extracted from a finite mixture model (Fig. 2a) were significantly different (KS-test, D = 0.21, *P* = 0.001), with the track azimuthal angles of the distribution to the left of the sun being larger than the azimuthal angles to the right (LRT, 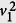 = 10.46, *P* = 0.001, Fig. 2b). In addition, the means of both distributions significantly differed from zero (Fig. 2b, left distribution (i.), (mean ± sd) −1.01 ± 0.05, LMM, t_126_ = 19.45, *P* < 0.001, right distribution (ii.), 0.59 ± 0.06, LMM, t_223_ = 19.75, *P* < 0.001) suggesting that subjects tended to move at angles to the sun, rather than directly towards/away from it. Although neither distribution significantly differed from a probabilistic normal distribution ((i.), KS test; D = 0.52, *P* = 0.977, (ii.), KS test; D = 0.72, *P* = 0.558), both were platykurtic displaying negative kurtosis values ((i.) = −1.25, (ii.) = −0.97). Examining these track azimuthal angles from the sun across observational periods, we observed that the angles changed in relation to the shifting positions of the sun in the sky (LRT, 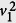 = 97.80, *P* = <0.001). In addition to this, relative azimuthal angular differences from the sun and tracks did not differ across observational periods suggesting subjects were moving at consistently similar offset angles throughout the day (LRT, 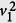 = 1.78, *P* = 0.619).

### How straight are tracks?

The angles of the tracks are only relevant if the subjects move in a near linear manner, i.e. move in a straight line from the start point to the stop point. The straightness index for the tracks was (mean ± sd) 0.89 ± 0.15 (see Supplementary Fig. S3), and the distribution of index values was heavily left skewed towards 1 (skewness value = −1.54).

### Caching and recovery times

**Figure 3:**
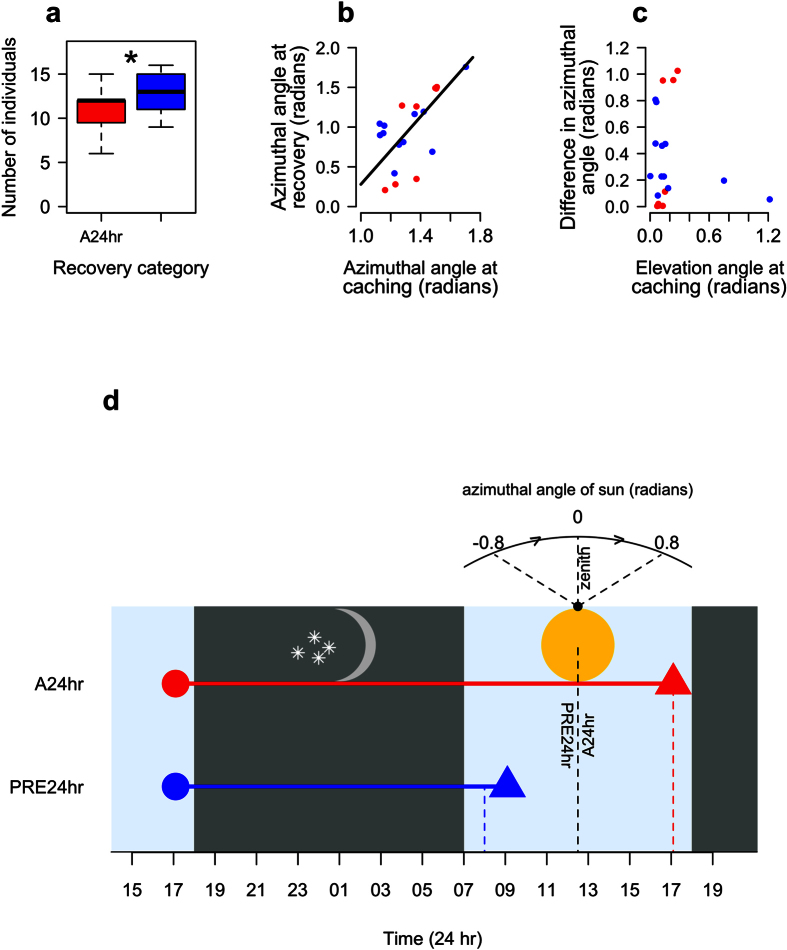
The influence of the number of individuals present in a group on the recovery period of the caches, (**a**). The relationship between the relative azimuthal angle of the sun at caching and recovery, (**b**) (The black line represents the predictions extracted during the modelling process). How the difference between the sun’s azimuthal angle at caching and recovery (proxy for accuracy) was affected by the elevation angle of the sun at caching, (**c**). Red points represent caches recovered within the A24 hr and blue points the PRE24 hr category. A diagram showing the time delay between caching (closed circles) and recovery (closed triangles) at the A24 hr (red) and PRE24 hr (blue) time points for 2 example caches, (**d**). The dashed coloured lines represent the solar azimuthal angles either 24-hours after the cache event (red) or at the opposite point in the solar arc to the cache event (blue). The black dashed line represents the zenith and the angular values above the plot correspond to the path of the sun’s arc.

The survival times of caches were negatively related to the number of individuals in a group, with more individuals leading to a reduction in the time until a cache was stolen (LRT, 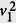 = 6.86, *P = *0.009). The recovery time for caches was 16.03 ± 5.84 (mean ± sd) hours, suggesting subjects were not solely relying on the position of the sun 24 hours later to recover. Caches were equally as likely to be recovered in the PRE24 hr and A24 hr categories (proportion tested = number of caches recovered in the PRE24 hr category: total number of caches recovered, proportion test: X^2^ = 0.45, *P* = 0.500). The decision about when to recover a cache may be influenced by a subject’s resident group size, where the number of individuals present was higher for when caches were recovered during the PRE24 hr as opposed to the A24 hr category (LRT, 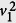 = 5.81, *P = *0.016, Fig. 3a). When subjects recovered within this PRE24 hr category, they did so when the sun was at the same azimuthal angle in the sky as the position during caching, but at the opposite point on its arc of trajectory (Fig. 1b). Where caches were recovered in the A24 hr category, they were more likely to be recovered near the 24-hour point. The relationship between these caching and relative recovery azimuthal angles of the sun was strongly significant (LRT, 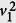 = 12.64, *P* < 0.001, Fig. 3b), suggesting subjects were recovering at the same relative elevation angle as during caching. The error, in terms of the differences in azimuthal angle at caching and recovery were not significantly related to elevation angles of the sun at caching (LRT, 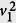 = 1.20, *P = *0.274, Fig. 3c). In addition no difference in error was observed between caches that were recovered in the PRE24 hr or A24 hr categories (LRT, 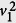 = 0.01, *P = *0.947).

### Size of foraging areas

The total null foraging area was significantly larger than the start point area, suggesting subjects were not homogenously found throughout a home range (LRT, 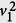 = 27.74, *P* < 0.005, mean % of total area covered by start points = 12.25 ± 7.18%, mean ± sd). This indicates that subjects foraged within a preferred area around the central burrow and showed a clustered distribution within the overall null area.

## Discussion

Although it is widely known that some animals rely on the sun for navigation, how they do this is still contentious^3^. Here we demonstrated that Cape ground squirrels might use the sun as a heading indicator, moving in near-linear lines, at consistent azimuthal offset angles from the sun. We suggest these behaviours allow individuals to gather information about the location of where they cached an item and reuse this information in recovering the food. As individuals have the ability to recover food within 24 hours, we propose that individuals show some flexibility in their reuse of the sun’s information. However, as this does not show a level of flexibility required for full time compensation, i.e., the ability to fully control for the shifting position of the sun throughout the diurnal period, we propose animals are reusing heading information from the sun in a time-limited way.

When Cape ground squirrels moved from start to stop points when caching food items, they appeared to move at azimuthal angles that were offset from the position of the sun, irrespective of whether they moved towards or away from the sun. We suggest that rather than making tracks with random angles in relation to the sun, subjects moved at consistent angles every time they placed a cache. Combined with the result that tracks were straight, the ground squirrels may be using simple rules, moving in near-linear directions at similar offset azimuthal angles from the sun to deposit a food item. These behaviours suggest that the sun was potentially being used as a heading indicator rather than a compass^3^, where individuals utilise the azimuthal angle of the sun, possibly in relation to other habitat features. The consistency of the track angles from the sun may allow for individuals to more easily obtain information about the track, rather than moving at random angles that would need to be memorised each time a cache was made. The reason for why these angles are offset from the absolute position of the sun may be due to solar glare, which could impact on an individual’s ability to monitor their environment^30^. In our example, monitoring the environment can refer to the ability to detect predators^30^,^31^ or gathering spatial information^32^ about where a cache is being located.

As Cape ground squirrels are central place foragers with preferred foraging patches we assumed that individuals would be familiar with landmark arrays around their home burrow. In homing pigeons, it was suggested that the mechanisms of foraging in unfamiliar areas are different from familiar areas^33^, where individuals may develop a “familiar area map”^34^. With these features, animals are predicted to develop a map based on memory of familiar landmarks. In addition, within such familiar areas, individuals may be able to learn how the path of the sun in the sky relates to landscape features, via associative learning processes^3^. We argue that in our study, subjects were using celestial information and memory of their foraging patch to locate the start point of a previous cache track. The endogenous circadian clock^35^ may allow for individuals to move to this start point at the appropriate time^3^,^36^,^37^, where they then reuse heading information from the sun to move to the cache site. We did not record the recovery tracks of individuals, yet this data would give better insight about the recovery reorientation behaviours and how similar they are to the caching behaviours.

Due to the sociality of this species, cache food stores are prone to high levels of theft^38^, and we found a large number of caches were stolen within 24 hours. This may lead to selection for mechanisms that allow animals to recover food within 24 hours. Theft of caches in social living animals has been linked to the number of individuals in a group, where random foraging movements of conspecifics may lead to competitors uncovering caches more rapidly^39^. In our study species, individuals have been shown to exhibit sensitivity to audience numbers by reducing the amount they cache, consuming the food item instead (J.S. unpublished data). In the current study, this fluctuation in competitor number explains why some caches were recovered before the 24-hour lag period. The ability to recover flexibly between the two time periods in response to an increase in competitor number could be driven by hormonal changes in the cacher. For example, cortisol has been shown to increase with group size^40^ and has also been implemented in causing changes in caching behaviour^41^.

The ability of animals to show full time-compensation in their use of the sun is thought to be mainly restricted to specialist navigators/orientators^3^,^16^, and therefore it is assumed not to occur within these squirrels. Rather, individuals may be reusing certain information from the sun in a ‘semi-flexible’ way, i.e. they are showing time dependency^7^ by recovering food around the 24-hour point, but additionally recovering food at a point pre-24 hours. When food was recovered within 24 hours, individuals were recovering when the sun was at the same azimuthal angle as the cache point, but on the opposite position of the suns arc. Individuals may be able to recover food at this point by compensating for the reversal of the suns position, potentially within a landmark array. Studies have shown how some species can account for a reversal in landmark arrays, by treating landmarks as units rather than individual elements^17^,^18^. However, how animals use this alternate position of the sun remains to be tested, as the animal’s endogenous circadian clock may only allow them to recover at 24 hours, i.e. in a strictly time dependent way. One suggestion is that the squirrels have a limited “solar ephemeris function”, which allows animals to control for the changing position of the sun throughout the day, where in this example, only the relative azimuthal angles of the sun are used^37^. The use of the sun in this way could suggest individuals show an adaptively specialised learning mechanism that is problem-specific^42^,^43^, where only fixed points on the solar ephemeris can be utilised. An alternative suggestion is that the squirrels may have a “matched filter” system, where groups of receptors in the cortex are matched to a spatial aspect of the orientational problem^44^. This system may account for the time-limited behaviours we observed in this study, as it does not require complex geometrical calculations. In our example receptors may be matched to the position of the sun during caching and the mirrored position of the sun at this point. However, as matched filter systems have only been described in invertebrates^44^,^45^, we can only speculate about the presence of such as system in the ground squirrels until further vertebrate models are studied.

Although we argue that Cape ground squirrels use the sun as a heading indicator whilst caching and recovering, a number of studies have suggested this cue has to be used in combination with other information, such as landmarks^4^,^13^. This study was undertaken in the squirrel’s natural habitat and therefore, manipulating landmarks was unfeasible. Additional experiments examining the interplay between landmark and celestial use would help us determine how individuals use these cues in combination to deposit and relocate caches. Furthermore, future laboratory studies using clock shift experiments, or controlling the availability of solar cues could further confirm that the sun is used for orientation in these squirrels. Animals appeared to be able to recover food items even with angular error in the position of the sun at recovery compared to the position at caching, suggesting additional cues may be used, such as landmarks. The “multiple bearing” hypothesis^17^ states that rather than an animal using a single cue and making others redundant, individuals can use a multitude of cues to increase spatial accuracy. In previous studies, orientational precision has been shown to decrease as the elevation angle of the sun increased towards the zenith^6^. In contrast, we observed no influence of elevation angle of the sun at caching on reorientation accuracy, possibly due to this amalgamation of multiple environmental cues reducing this error. However, as we did not have a large sample size of different elevation angles throughout the day, we cannot rule out that this species has a similar precision problem as shown in other studies.

Although discussed within the context of caching behaviour only, the evolution of such an orientation system could also be linked to general foraging behaviour. In the Kalahari, food is dispersed and sparse, but occasionally bonanza resources are located and these are often clumped. For example, bulbs of the bushveld vlei lily (*Nerine laticoma*) and the fruiting body of the tsamma melon (*Citrullus lanatus*) are sporadically uncovered or sprout within the foraging range of a group. Therefore, it may be beneficial for an individual to memorise the location of such a patch, which can then be visited on successive foraging forays^46^. The mechanism by which the location of these patches could be acquired would be similar to what is described for caching behaviour, whereby individuals utilise cues from the sun and possible landmarks that would enable individuals to re-orientate back to that patch.

To our knowledge this is the first study on wild mammals to describe such strategic use of the sun in this way. In general, our study sheds light on the interplay between time-compensated and time-dependent use of the sun as a heading indicator. We suggest that this flexibility may have evolved due to the high risk of caches being stolen, and animals have developed a problem specific strategy. The time-limited use of the sun identified in these squirrels may encourage other studies on species that display time-dependency to examine to what degree this behaviour is flexible, and how it is manifested in subsequent behaviour.

## Additional Information

**How to cite this article**: Samson, J. and Manser, M. B. Use of the sun as a heading indicator when caching and recovering in a wild rodent. *Sci. Rep.* **6**, 32570; doi: 10.1038/srep32570 (2016).

## Supplementary Material

Supplementary Information
