# Supplementary material for: Use of the sun as a heading indicator when caching and recovering in a wild rodent
Source: Sci Rep. 2016 Sep 1;6:32570. doi: 10.1038/srep32570 (PMC5007651; doi:10.1038/srep32570)
Supplement: Supplementary Information [file srep32570-s1.pdf]

Supplementary information

for

Use of the sun as a heading indicator when caching and recovering in a wild rodent

Jamie Samson[1]* & Marta B. Manser[1]

[1]Department of Evolutionary Biology and Environmental Studies, University of Zurich, Switzerland

*Corresponding author: Jamie Samson

Department of Evolutionary Biology and Environmental Studies, University of Zurich, Winterthurerstrasse 190, 8057, Zurich, Switzerland

Email: jamie.samson@ieu.uzh.ch

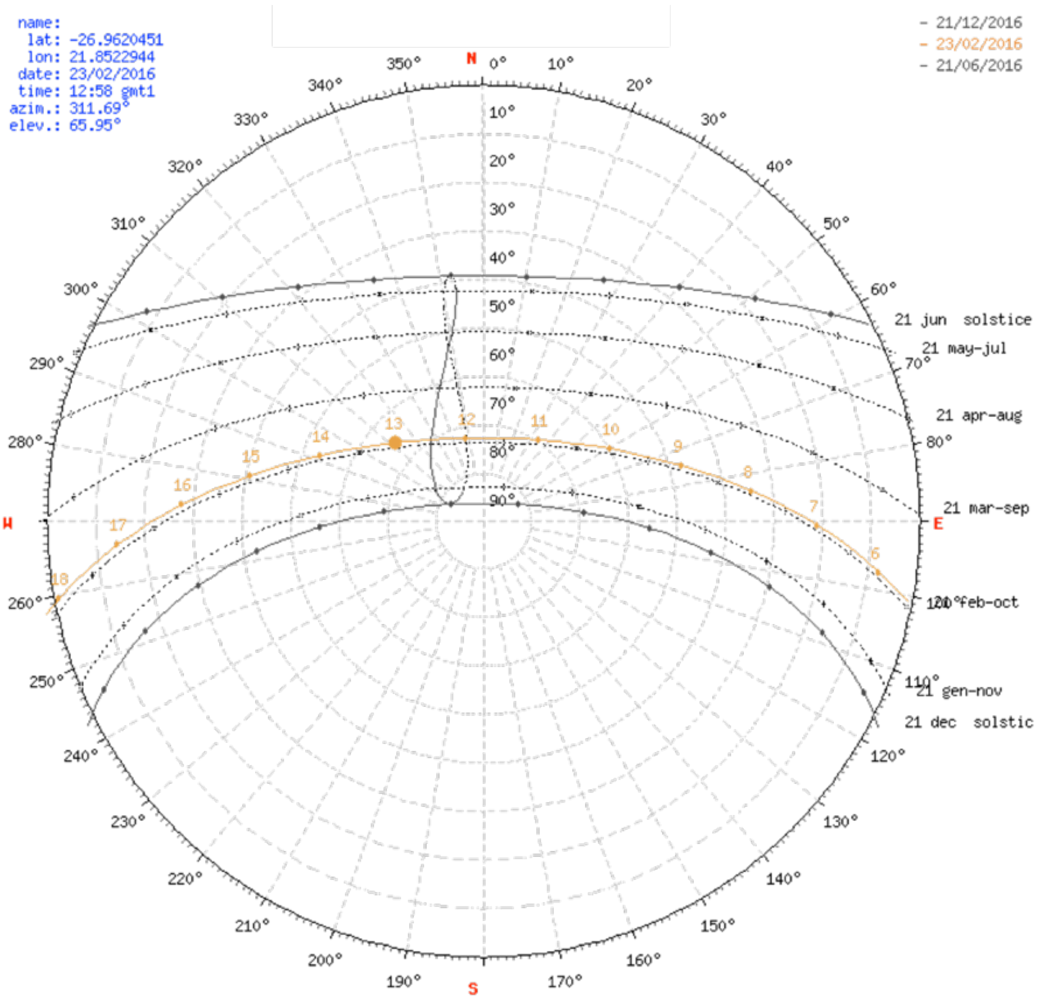

Figure S1: An example of the diagrams extracted from the SunEarthTools repository. For each group, we used the coordinates of the centre of the sleeping burrow to obtain the local path of the sun in the sky. In addition, we used the precise time and date of the track to obtain an accurate measurement of the azimuthal angle of the sun at this time.

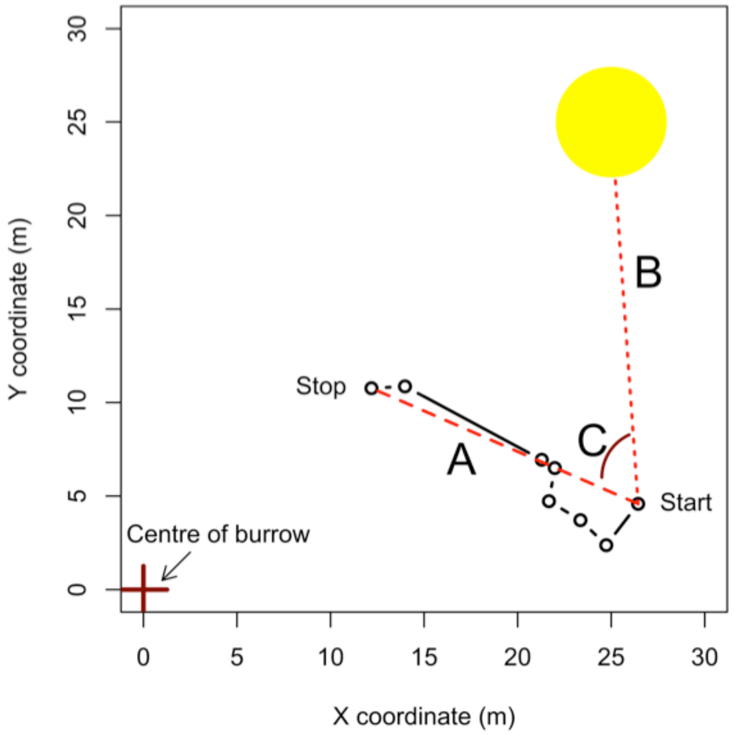

Figure S2: How the azimuthal angle (C) between the sun (B) and track (A) was calculated. The local sun diagrams from Fig. S1 were overlaid on the groups home range and then both the angle of the sun and the points of the track were calculated in relation to the centre of the home range (0,0). As tracks were significantly straight in form, we were justified in using the Euclidean distance between the start and stop points of the tracks to allow for the calculation of the azimuthal angle.

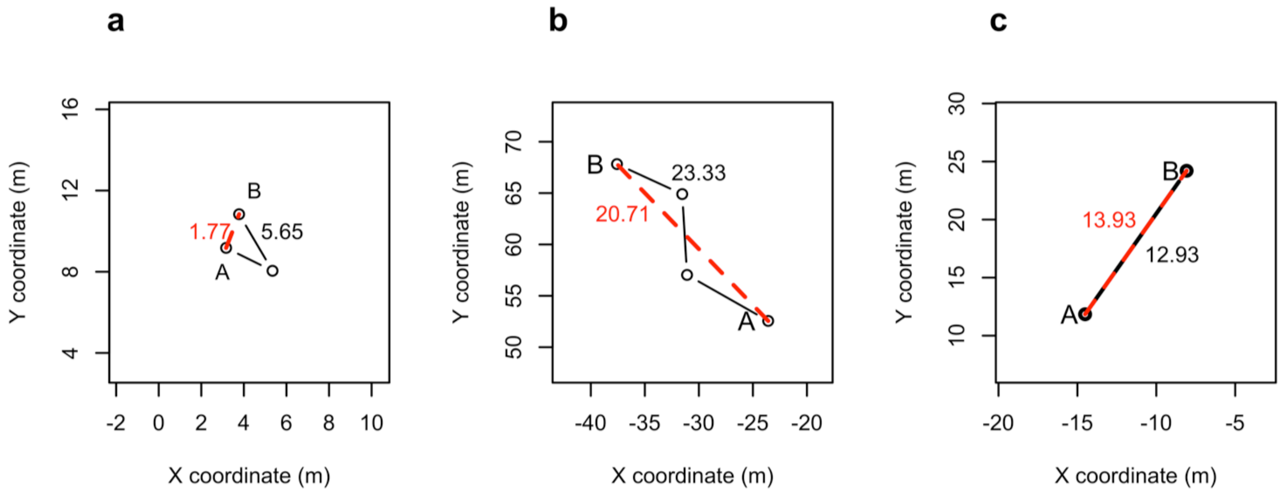

Figure S3: Examples of tracks of varying lengths (lengths, in metres, donated by the numbers) and straightness; highly tortuous (a), average tortuosity (b), straight (c). The tortuosity was calculated using the "straightness index"[1], which is the Euclidean distance (red) divided by the observed distance of the tracks (black).

Table S1: Details of the linear mixed effects models used in this study. The significance of the fixed effects in these models was determined using likelihood ratio tests unless otherwise stated.

Model variables		Model output				Likelihood ratio test		
Predictor	Response	Estimate	SE	t	*P*	ν^2	*P*	Figure
Azimuthal angle from sun (all tracks)	Distribution (2 levels, to the left or right)	-0.15	0.05	-3.26		10.46	**< 0.01**	2.
Azimuthal angle from sun (left)	1 (difference from 0)*	0.85	0.04	19.45	**< 0.001**			2.
Azimuthal angle from sun (right)	1 (difference from 0)*	0.7	0.04	19.75	**< 0.001**			2.
Azimuthal angle of sun	Azimuthal angle from sun (all tracks)	0.85	0.08	11.28		97.8	**< 0.001**	
Azimuthal angle from sun (all tracks)	Observation period (4 levels, observation period 1,2,3,4)‡					1.78	0.619	
Survival time (hours)	Number of individuals present in a group	-0.13	0.05	-2.8		6.87	**< 0.01**	
Number of individuals present in a group	Recovery period (2 levels, PRE24hr or A24hr)	1.71	0.69	2.48		5.81	**0.015**	3a.
Azimuthal angle of sun (recovery)	Azimuthal angle of sun (caching)	2.13	0.48	4.47		12.64	**< 0.001**	3b.
Elevation angle of the sun	Difference between azimuthal angle at caching and recovery	-1.07	0.5	-2.13		4.48	**0.034**	3c.
Difference between azimuthal angle at caching and recovery	Recovery period (2 levels, PRE24hr or A24hr)	-0.03	0.16	-1.92		0.05	0.818	

**p value calculated from the model output rather than via likelihood ratio testing as models only contained a random fixed effect (1). This was used to determine the difference between the azimuthal angles of tracks to the left and right of the sun from zero (i.e. the azimuthal angle of the sun).*

‡*As we were only concerned with the difference across observational periods, we did not present model outputs for each comparison.*

Supplementary references:

S1. Batschelet, E. *Circular Statistics in Biology* (Academic Press, 1981).
